# Supplementary material for: Solar ultraviolet B radiation promotes α-MSH secretion to attenuate the function of ILC2s via the pituitary–lung axis
Source: Nat Commun. 2023 Sep 12;14:5601. doi: 10.1038/s41467-023-41319-1 (PMC10497598; doi:10.1038/s41467-023-41319-1)
Supplement: Supplementary file 1 — Supplementary Information [file 41467_2023_41319_MOESM1_ESM.pdf]

Solar ultraviolet B radiation promotes α-MSH secretion to attenuate the function of ILC2s via the pituitary–lung axis

Supplementary Figure

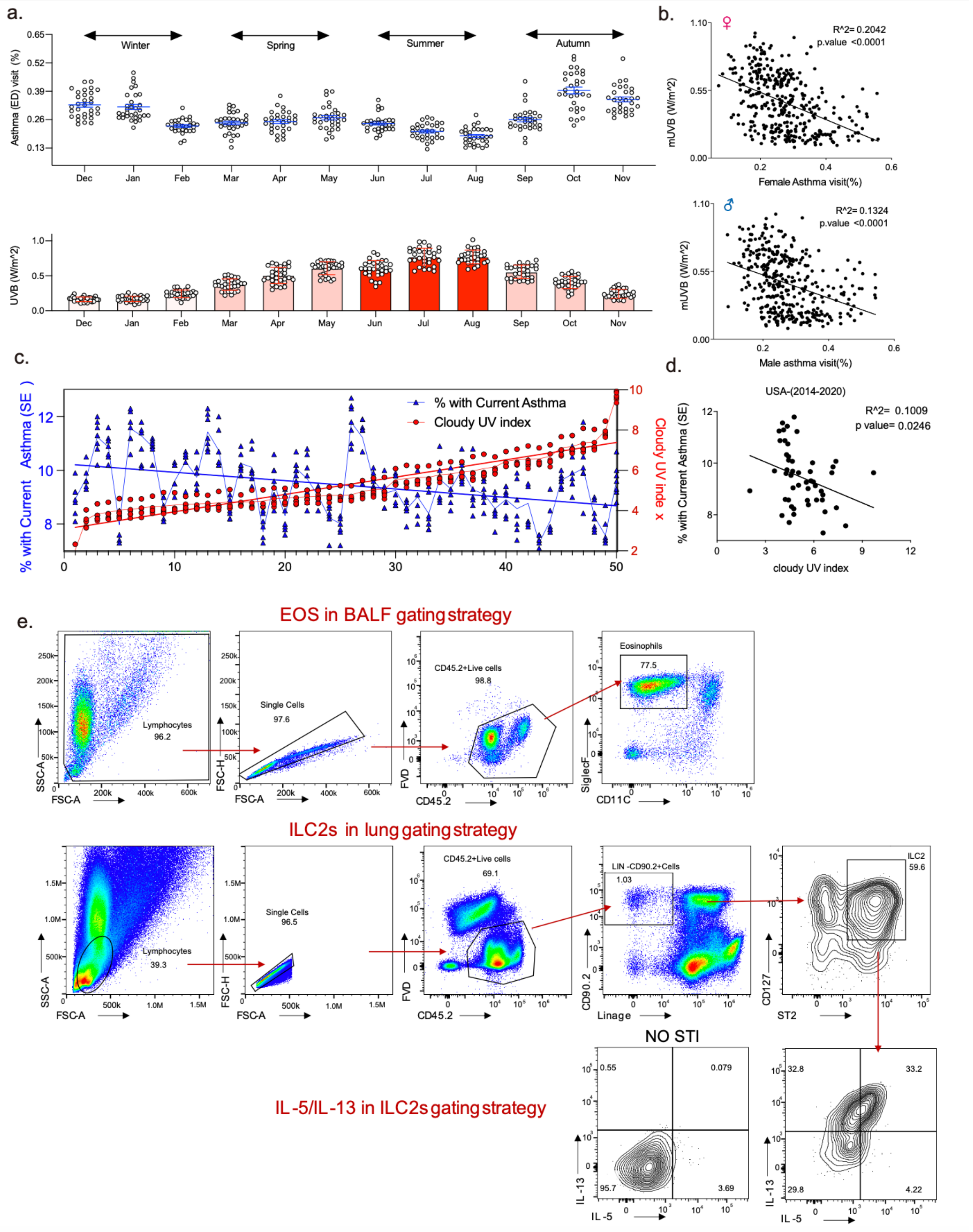

Supplementary Figure1. Solar exposure decreased the asthma visit rate.

(**a**) Dot plot of the average asthma visit distribution (daily asthma visit counts/yearly asthma visit counts, n=days of current month) from 2015 to 2019 for 18,431 people in Shanghai. Lower panel: Monthly average UVB radiation (W/m^2) from 2015 to 2019 in Shanghai (n=31,31,28,31,30,31,30,31,31,30,31,30 from left to right); the red intensity reflects the radiation strength (n=31,31,28,31,30,31,30,31,31,30,31,30 from left to right). (**b**) Correlation analysis between the average female(n=8,006) or male(n=10,425) asthma visit rate (daily average) and corresponding UVB radiation from 2015 to 2019 in Shanghai. (**c**) Dot plot showing the yearly asthma morbidity (blue line, data from the Centers for Disease Control and Prevention) and the yearly average UV index (red line, data from the Climate Prediction Center) from 2014 to 2020 in the USA. (**d**) Correlation analysis of the percentage of current asthma and the corresponding UV index from 2014 to 2020 in the USA. (**e**)Gating strategy of eosinophils in BALF (Eosinophils, Eos; $FVD^-CD45^+CD11c^{-/lo}SiglecF^+$), ILC2s (ILC2s, $FVD^-CD45.2^+LIN^-CD90.2^+CD127^+ST2^+$) in lung, IL-5+/IL-13+in ILC2s ($FVD^-CD45.2^+LIN^-CD90.2^+CD127^+ST2^+IL\text{-}5^+IL\text{-}13^+$), IL-5 and IL-13 in lung ILC2s stimulated with PMA plus ionomycin and BFA for 4 h. The bars and error bars show the means ± SDs. Pearson’s correlation coefficient analysis was used.

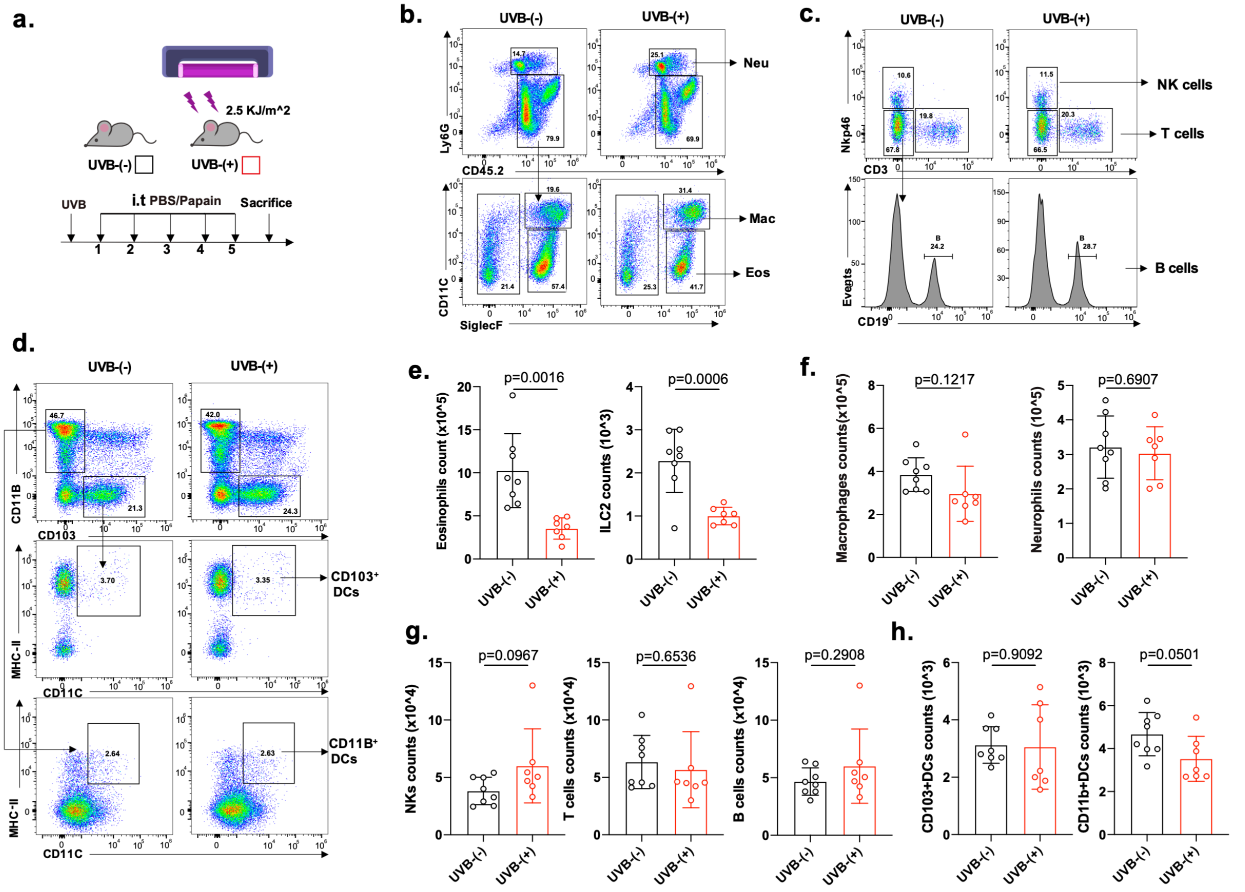

Supplementary Figure2. Myeloid and lymphoid cell distributions in the lungs of mice after exposure to UVB.

(**a**) Schematic diagram of the experimental design. WT female mice were intratracheally challenged with papain (5 µg per mouse) for 5 consecutive days, and UVB radiation (2.5 kJ/m^2) exposure occurred 1 day before sensitization. The mice were sacrificed on day 6. (**b**) Gating strategy for lung neutrophils (Neu: $FVD^{-}CD45^{+}Ly6G^{+}$), eosinophils (Eos: $FVD^{-}CD45^{+}Ly6G^{-}Siglec\text{-}F^{+}CD11c^{-}$), macrophages (MAC: $FVD^{-}CD45^{+}Ly6G^{-}SiglecF^{+}CD11c^{+}$).(**c**) Gating strategy for lung natural killer (NK) cells ($FVD^{-}CD45^{+}CD3^{-}NKp46^{+}$), T cells ($FVD^{-}CD45^{+}CD3^{+}$), and B cells ($FVD^{-}CD45^{+}CD3^{-}NKp46^{-}CD19^{+}$).(**d**) Gating strategy for lung $CD103^{+}$ DCs ($FVD^{-}CD45^{+}CD11b^{-}CD103^{+}CD11c^{+}$MHC class II^{+}) and $CD11b^{+}$ DCs ($FVD^{-}CD45^{+}CD11b^{+}CD103^{-}CD11c^{+}$MHC class II^{+}). (**e**) The number of eosinophils and ILC2s in lung from papain challenged mice with or without UVB treatment(n=8,7). (**f**) Number of macrophages and neutrophils in lung from papain challenged mice with or without UVB treatment(n=8,7). (**g**) Number of NKs, T cells, B cells in lung from papain challenged mice with or without UVB treatment(n=8,7). (**h**) Number of $CD103^{+}$ DCs and $CD11b^{+}$ DCs in lung from papain challenged mice with or without UVB treatment(n=8,7). Each symbol represents an individual mouse(**e,f,g,h**). Data are representative of two or more independent experiments. The bars and error bars show the means ± SDs. **e,f,g,h**, two-tailed unpaired Student's t test.

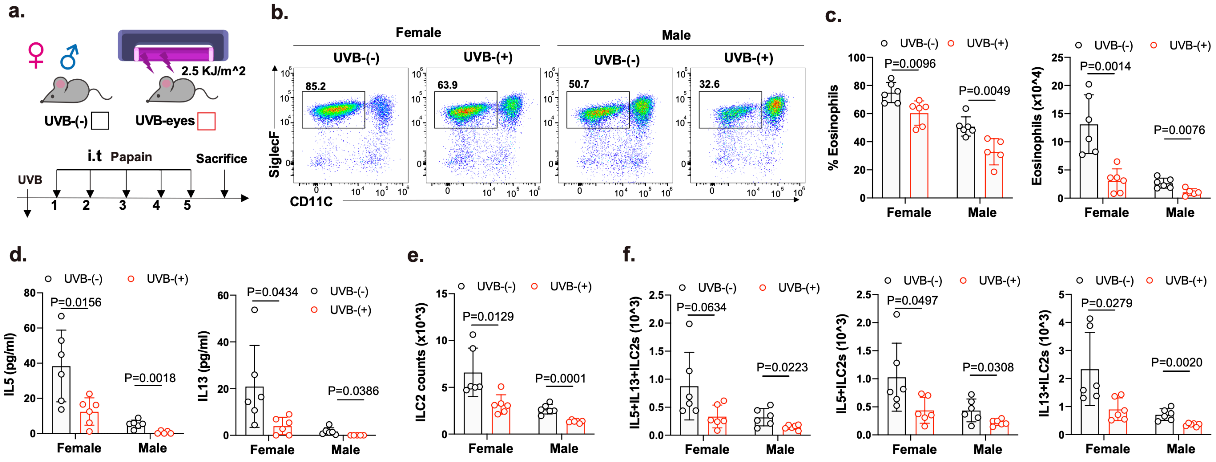

Supplementary Figure 3. UVB exposure suppressed papain-induced lung inflammation both in female and male mice.

(**a**) Schematic diagram of the experimental design. WT female and male mice were intratracheally challenged with papain (5 µg per mouse) or PBS for 5 consecutive days, and UVB radiation (2.5 kJ/m2) exposure occurred 1 day before sensitization. The mice were sacrificed on day 6. (**b-c**) Percentage and number of eosinophils in the BALF (Eosinophils, Eos; FVD$^-$CD45$^+$CD11c$^{-/lo}$SiglecF$^+$) (n=6/group). (**d**) The levels of IL-5 and IL-13 in the BALF were measured by ELISA(n=6/group). (**e**) Statistical analysis of the numbers of ILC2s in the lungs (ILC2s, FVD$^-$CD45.2$^+$LIN$^-$CD90.2$^+$CD127$^+$ST2$^+$) (n=6/group). (**f**) Statistical analysis of the numbers of IL-5$^+$IL-13$^+$ILC2s, IL-5$^+$ILC2s, IL-13$^+$ILC2s stimulated with PMA plus ionomycin and BFA for 4 h(n=6/group). Each symbol represents an individual mouse(**c,d,e,f**). Data are representative of two or more independent experiments. The bars and error bars show the means ± SDs. **c,d,e,f,** two-tailed Student's t test were used.

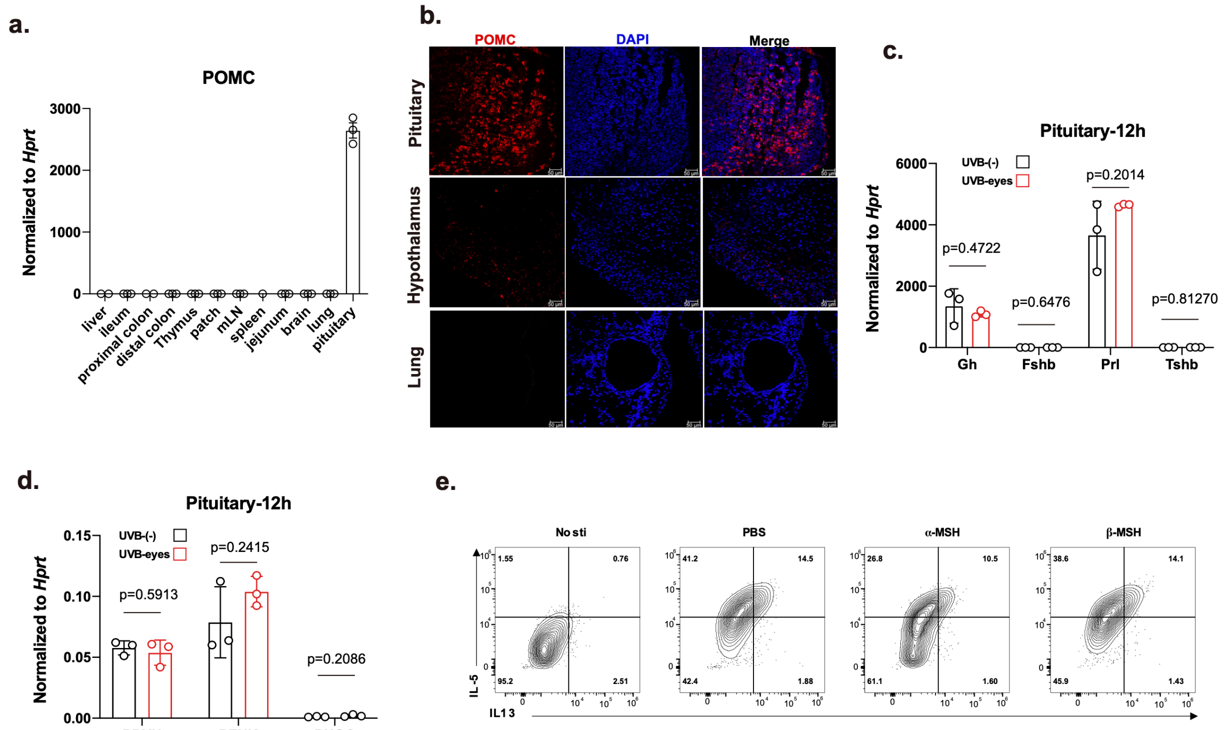

Supplementary Figure 4. Expression of the α-MSH precursor POMC.

(**a**) qRT–PCR analysis of *Pomc* expression in various tissues (n=3/group). (**b**) Confocal analysis of POMC expression in the pituitary gland, hypothalamus, and lungs. (**c**) qRT–PCR analysis of *Gh*, *Fshb*, *Prl*, *Tshb*, and *Pomc* expression in the pituitary gland (n=3/group). (**d**) qRT–PCR analysis of *Pydn*, *Penk*, *Ponc*, and *Pomc* expression in the pituitary gland (n=3/group). (**e**) Flow cytometric analysis of IL-5 and IL-13 in lung ILC2s stimulated with PMA plus ionomycin and BFA for 4 h. **a,c,d,** Gene expression was analyzed by qRT-PCR and normalized with *Hprt* using $2^{-\triangle\triangle Ct}$ method. Data are representative of two or more independent experiments. Each symbol represents an individual mouse(**a,c,d**).The bars and error bars show the means ± SDs. **c,d**, two-tailed Student's t test were used.

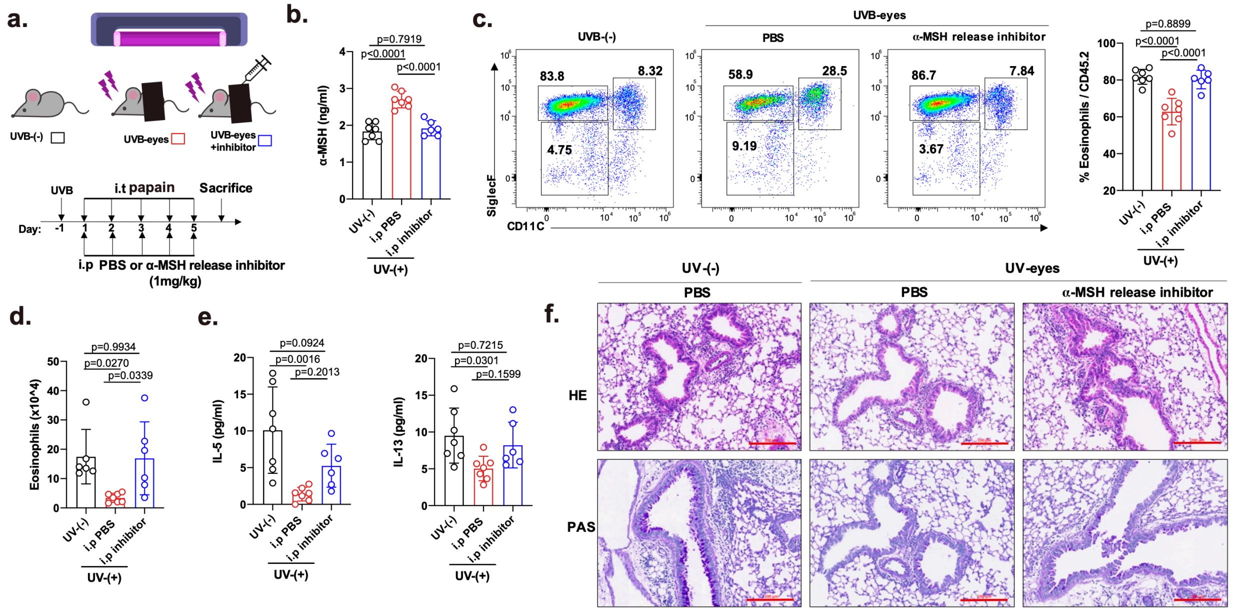

Supplementary Figure 5. An α-MSH release inhibitor rescued the inhibitory effect of UVB radiation on ILC2-mediated lung inflammation.

(**a**) Schematic diagram of the experimental design. WT female mice were challenged with papain and treated with an α-MSH release inhibitor for 5 consecutive days. (**b**) The levels of α-MSH in the serum were measured by ELISA (n=7,7,6 from left to right). (**c**) The percentage of eosinophils in the BALF (n=7,7,6 from left to right) (n=7,7,6 from left to right). (**d**) The number of eosinophils in the BALF (n=6,7,6 from left to right). (**e**) The levels of IL-5 and IL-13 in the BALF were measured by ELISA (n=7,7,6 from left to right). (**f**) Representative H&E and PAS staining of lung sections (bars, 200 μm)(n=6/group). Each symbol represents an individual mouse(**b,c,d,e**). Data are representative of two or more independent experiments. The error bars show the means ± SDs. **b,c,d,e,** one-way ANOVA with Tukey's multiple comparisons test.

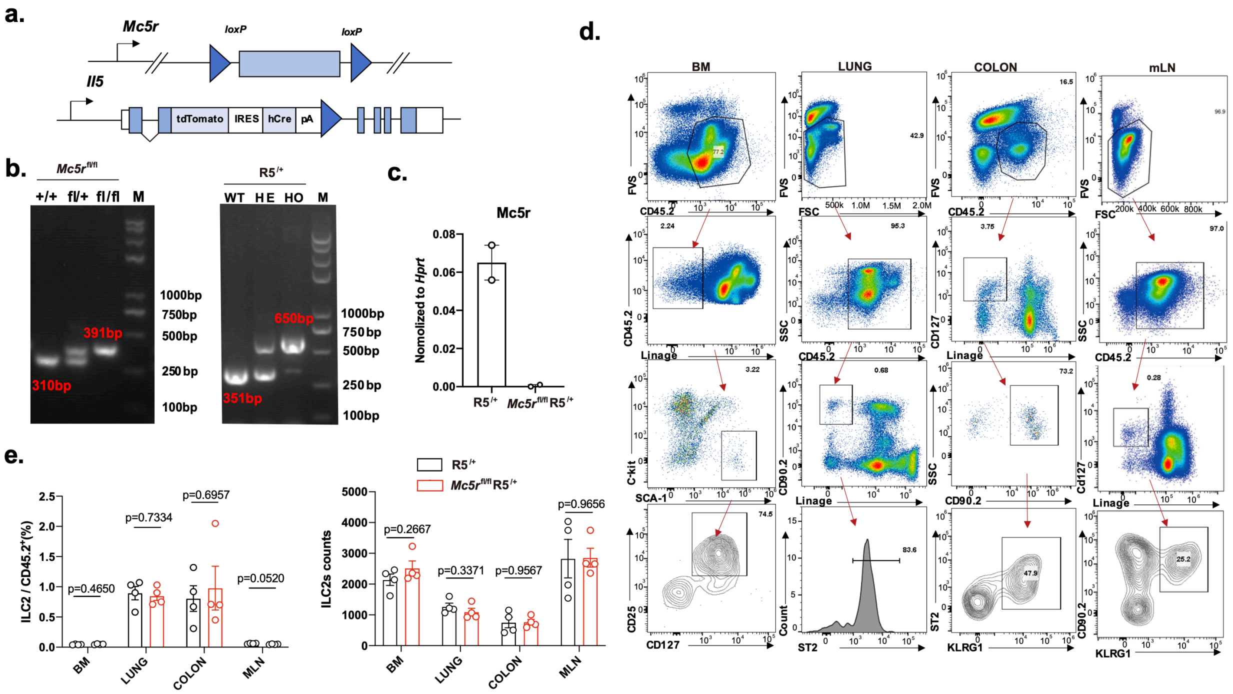

Supplementary Figure 6. ILC2 function was unperturbed in *Mc5r*$^{fl/fl}$R5$^{/+}$ mice in the steady state.

(**a**) Schematic of targeting construct. (**b**)Scans of the genotyping of R5$^{/+}$ and *Mc5r*$^{fl/fl}$R5$^{/+}$ female mice. (**c**) qRT–PCR analysis of Mc5r expression in lung ILC2s of R5$^{/+}$ and *Mc5r*$^{fl/fl}$R5$^{/+}$ female mice(n=2/group). Gene expression was analyzed by qRT-PCR and normalized with HPRT using $2^{-\triangle\triangle Ct}$ method. (**d**) Flow cytometry plots of ILC2s in the BM (FVD$^-$CD45.2$^+$Lin$^-$C-kit$^+$Sca1$^+$CD25$^+$CD127$^+$), lungs (FVD$^-$CD45.2$^+$Lin$^-$CD127$^+$CD90.2$^+$ST2$^+$), colon (FVD$^-$CD45.2$^+$Lin$^-$CD127$^+$CD90.2$^+$ST2$^+$KLRG1$^+$), and mLNs (CD45.2$^+$Lin$^-$CD127$^+$CD90.2$^+$KLRG1$^+$) of R5$^{/+}$ and *Mc5r*$^{fl/fl}$R5$^{/+}$ female mice in the steady state. (**e**) Left: Statistical analysis of the percentage of ILC2s in the BM, lungs, colon and mLNs (n=4/group); Right:Statistical analysis of the numbers of ILC2s in the BM, lungs, colon and mLNs (n=4/group). Each symbol represents an individual mouse(**c,e**). Data are representative of two independent experiments. The bars and error bars show the means ± SEMs. **c,e,** two-tailed unpaired Student's t test were used.

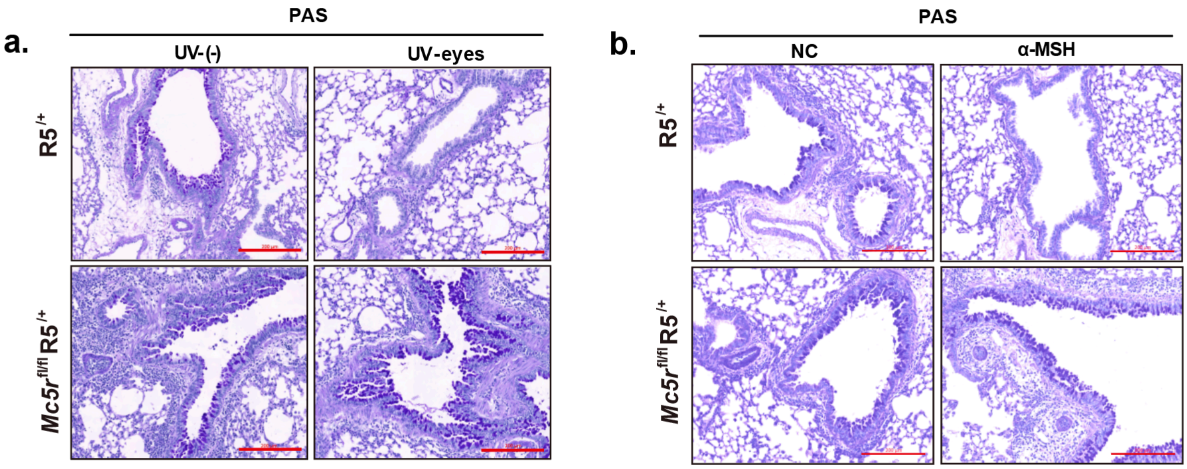

Supplementary Figure 7. UVB radiation inhibits lung inflammation via MC5R on ILC2s, related to Fig. 3.

(**a**) Representative PAS staining of lung sections (bars, 200 μm), related to Fig3c. (**b**) Representative PAS staining of lung sections (bars, 200 μm), related to Fig3i.

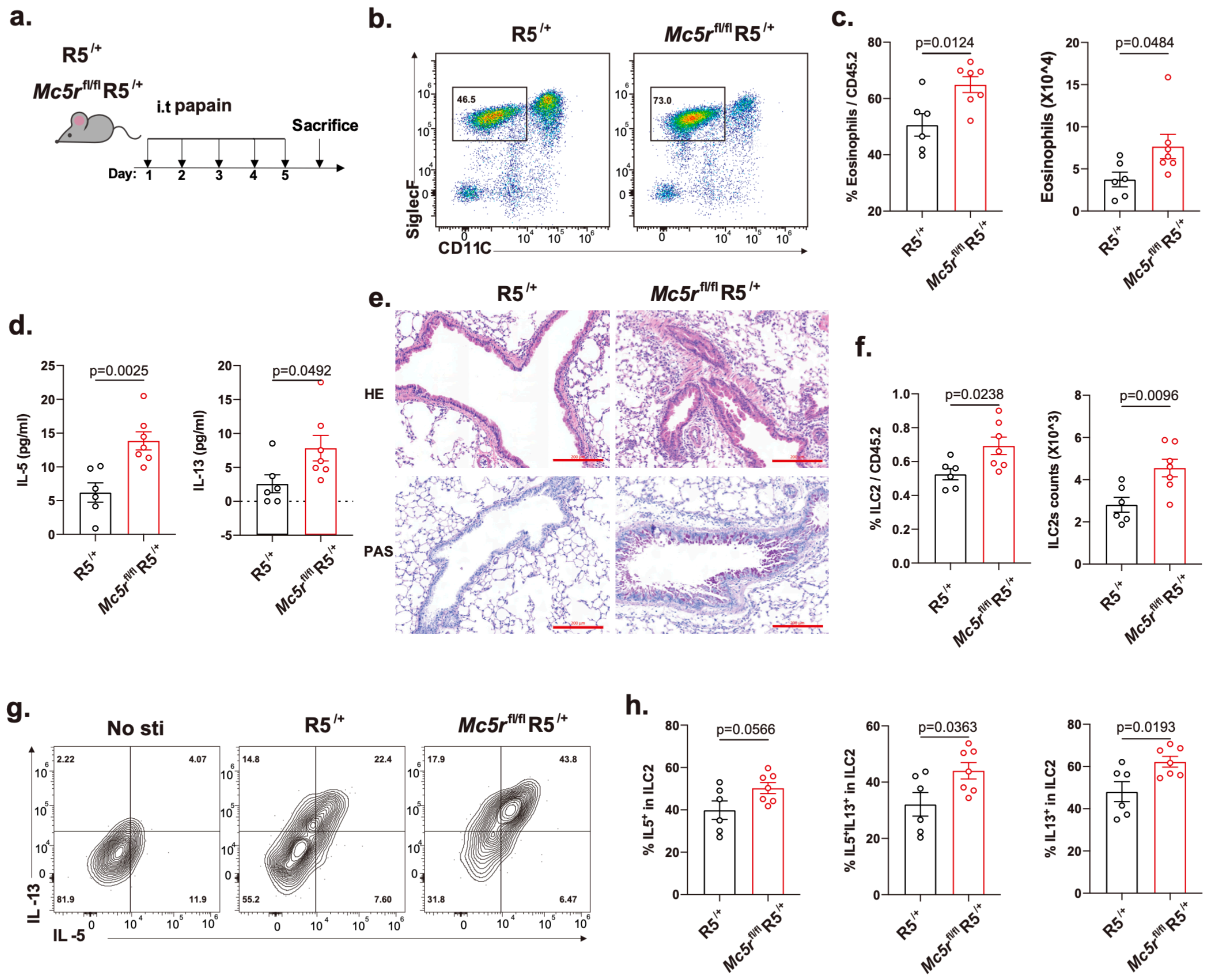

Supplementary Figure 8. *Mc5r*$^{fl/fl}$R5$^{/+}$ mice exhibited exacerbated lung inflammation and increased ILC2s.

(**a**) Schematic diagram of the experimental design. R5$^{/+}$ and *Mc5r*$^{fl/fl}$R5$^{/+}$ female mice were intratracheally challenged with papain (4 µg per mouse) for 5 consecutive days and then sacrificed on day 6. (**b**) Flow cytometry plots for eosinophils among live CD45^{+} cells in the BALF. (**c**) Percentage and number of eosinophils in the BALF (n= 6,7). (**d**) ELISA was performed to measure the levels of IL-5 and IL-13 in the BALF (n= 6,7). (**e**) Representative H&E and PAS staining of lung sections (bars, 200 µm). (**f**) Statistical analysis of the percentage and numbers of ILC2s in the lungs(n= 6,7). (**g**) Representative flow diagram showing IL-5 and IL-13 in lung ILC2s stimulated with PMA plus ionomycin and BFA for 4 h. (**h**) Statistical analysis of the percentages of IL-5^{+} and IL-13^{+} cells among lung ILC2s stimulated with PMA plus ionomycin and BFA for 4 h (n= 6,7). Each symbol represents an individual mouse(**c,d,f,h**).The bars and error bars show the means ± SEMs. **c,d,f,h,** two-tailed unpaired Student's t test.

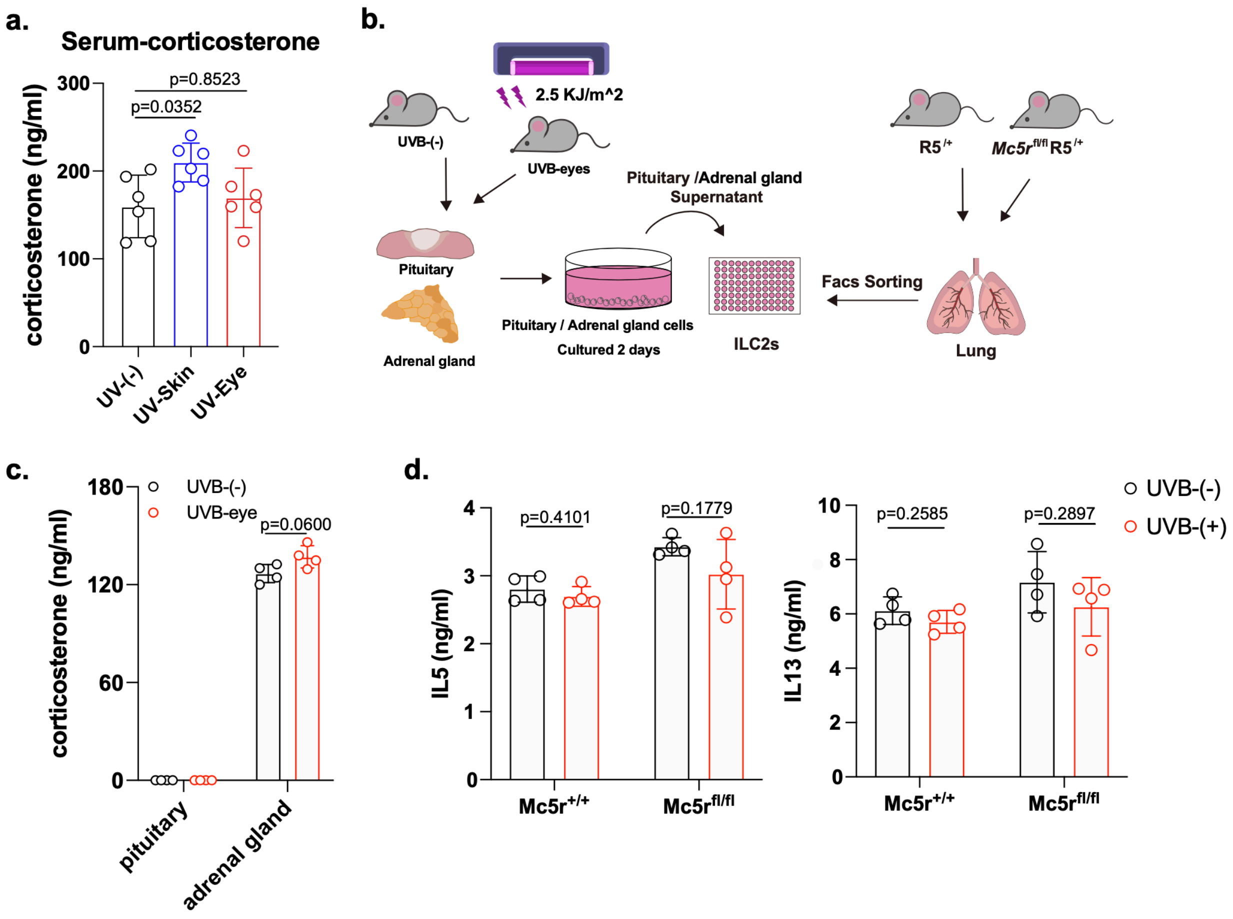

Supplementary Figure 9. Corticosterone concentration and biological activity in UVB-eye axis.

(**a**) The levels of corticosterone in the serum were measured by ELISA 12h after UVB-eye radiation (n= 6/group). (**b**) Schematic diagram of the experimental design: ILC2s from $R5^{/+}$ and $Mc5r^{fl/fl}R5^{/+}$ female mice cultured with the culture supernatant of pituitary cells or adrenal gland cells from WT mice treated with or without UVB irradiation to the eyes. (**c**) The levels of corticosterone in the pituitary cell or adrenal gland cell culture supernatant were measured by ELISA (n= 4 per group). (**d**) The IL-5 and IL-13 protein levels in the supernatants were detected by ELISA (n=4 per sample). Each symbol represents an individual mouse (**a,c,d**). The data are representative two or more independent experiments. The bars and error bars show the means ± SDs. For statistical analysis, the following tests were used. **a,**one-way ANOVA with Tukey's multiple comparisons test. **c,d,** two-tailed unpaired Student's t-test.

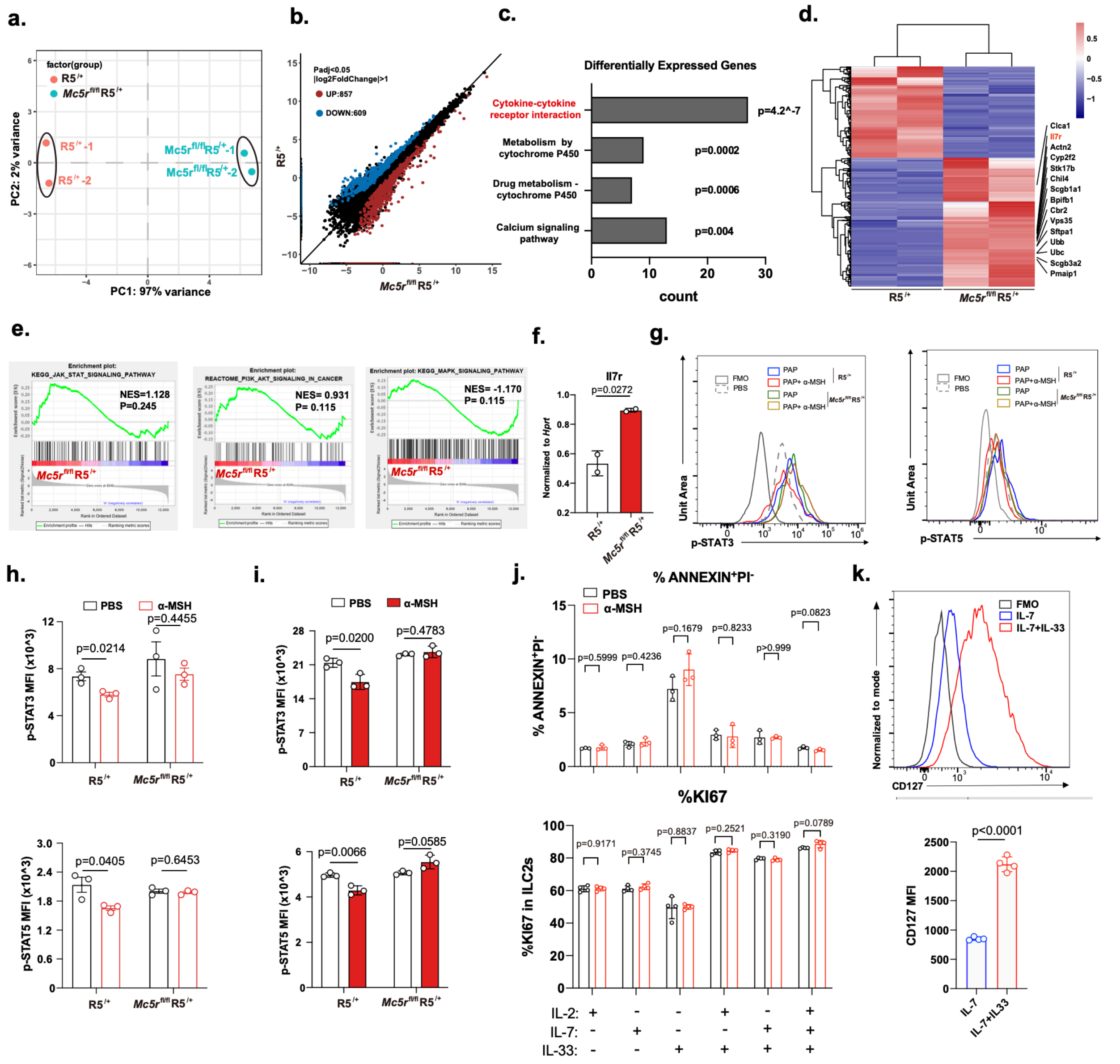

Supplementary Figure 10. α-MSH inhibits ILC2 function through the JAK/STAT signalling pathway.

(**a**) Principal component analysis (PCA). (**b**) Volcano plot of differentially expressed genes (log2(fold change) > 1.0; P < 0.05) in ILC2s compared between *R5*$^{/+}$and *Mc5r*$^{fl/fl}$R5$^{/+}$ mice. Upregulated genes and downregulated genes are highlighted in blue and red. (**c**) Kyoto Encyclopedia of Genes and Genomes analysis. (**d**) Heatmap of the differentially expressed genes. (**e**) GSEA of the JAK/STAT, PI3K/AKT, and MAPK signalling pathways. (**f**) qRT–PCR analysis of *Il7r* in ILC2s from *R5*$^{/+}$ and *Mc5r*$^{fl/fl}$R5$^{/+}$ mice (n=2 mice/group). (**g**) *R5*$^{/+}$ and *Mc5r*$^{fl/fl}$R5$^{/+}$ female mice were treated with papain or papain plus α-MSH, and the levels of p-STAT3 and p-STAT5 in lung ILC2s were determined by flow cytometry. (**h**) Statistical analysis of the mean fluorescence intensities (MFIs) of p-STAT3 and p-STAT5 in **g** (n=3 mice/group). (**i**) Statistical flow cytometric analysis of the MFIs of p-STAT3 and p-STAT5 in sorted ILC2s from *R5*$^{/+}$ and

*Mc5r^{fl/fl}*R5^{/+} female mice in vitro (n=3 /group). (**j**) Flow cytometric analysis of PI^{-}Annexin V^{+}ILC2s after 3 days treatment of α-MSH. Flow cytometric analysis of PI^{-}Annexin V^{+}ILC2s after 3 days treatment of α-MSH (n=3/group). ILC2s cultured with IL-2 (100 U/ml), IL-7(20 ng/ml), IL-33(1 ng/ml) respectively, and combinations of IL-2 (100 U/ml) plus IL-33(1 ng/ml), IL-7(20 ng/ml) plus IL-33(1 ng/ml) or IL-2 (100 U/ml) plus IL-7(20 ng/ml) and IL-33(1 ng/ml) in the context of α-MSH treatment (n=4/group). (**k**) Flow cytometric analysis of CD127 in ILC2s under the different described experimental conditions(n=4/group). Each symbol represents an individual mouse (**f,h**) or samples(**i,j,k**). The data are representative of two or more independent experiments. The bars and error bars show the means ± SDs. **f,h,i,j,k,** two-tailed unpaired Student's t-test.

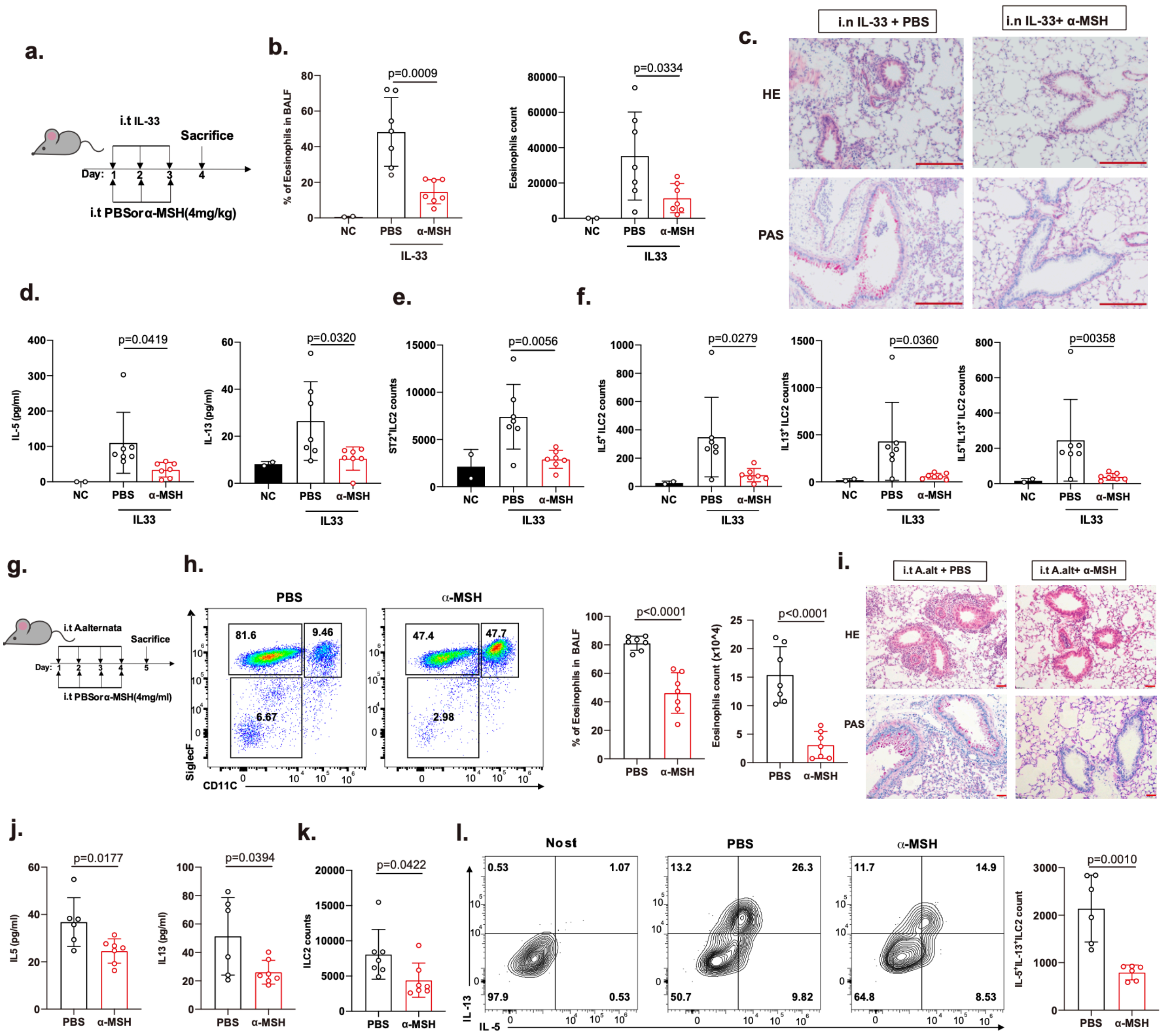

Supplementary Figure 11. Administration of α-MSH ameliorated ILC2 responses in IL-33/ *A. alternata*–induced type 2 immune response.

(**a**) Schematic diagram of α-MSH treatment through intratracheal delivery in an IL-33 induced type 2 immune response. (**b**) The percentage and number of eosinophils in BALF (n=2,7,7 from left to right). (**c**) Representative H&E and PAS staining of lung sections (bars,200μm). (**d**) The levels of IL-5 and IL-13 in BALF were measured by ELISA (n=2,7,7 from left to right). (**e**) The number of lung ILC2s (n=2,7,7 from left to right). (**f**) The absolute number of ILC2s secreting IL-5 and IL-13(n=2,7,7 from left to right). (**g**) WT female mice were intratracheally challenged with *A. alternata* (4 μg per mouse) or PBS for 4 consecutive days, treated with PBS or α-MSH (4 mg/kg) daily and sacrificed on day 5. (**h**) Quantification of total eosinophils in BALF (n =7/ group). (**i**) Representative H&E and PAS staining of lung sections (bars, 50 μm, n =7/group). (**j**) The levels of IL-5 and IL-13 in BALF were measured by ELISA (n=6,7 from left to right). (**k**) Statistical analysis of the numbers of ILC2s in the lungs (n =7/group). (**l**) Flow cytometric analysis of IL-5 and IL-13 in lung ILC2s stimulated with PMA plus ionomycin and BFA for 4 h (n =6/group). Each symbol represents an individual mouse(**b,d,e,f,h,j,k,l**).The data are representative of two or more independent experiments. The bars and error bars show the means ± SEMs. For statistical analysis,

the following tests were used. **b,d,e,f,** one-way ANOVA with Tukey’s multiple comparisons test. **h,j,k,l**, two-tailed unpaired Student's t-test.

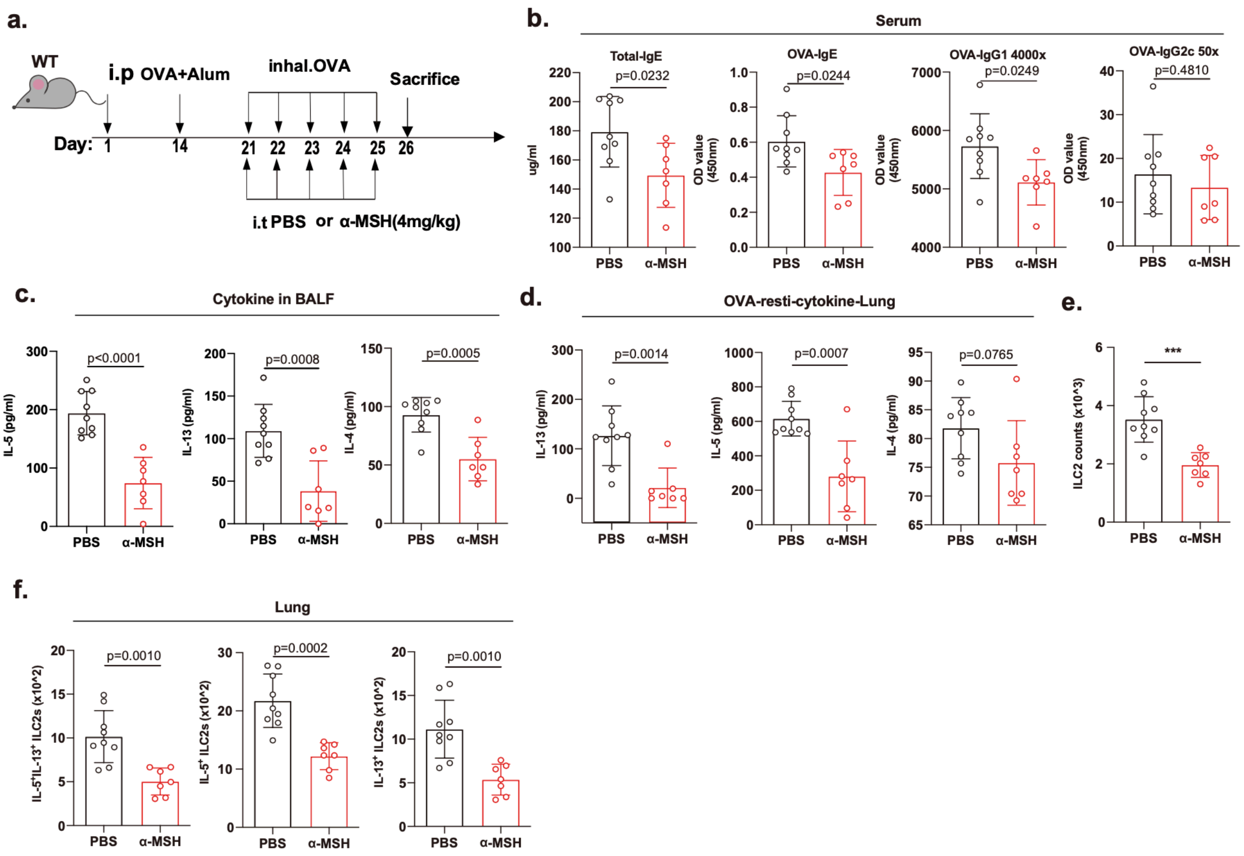

Supplementary Figure 12. The role of α-MSH in OVA- induced chronic lung inflammation.

(**a**) WT female mice were primed with an intraperitoneal injection of 20 μg of OVA-alum in 200 μl on days 1 and 14. On days 21–25, 10 mg/ml OVA in PBS was administered to the mice by inhalation. On day 26, the mice were sacrificed. (**b**) The total IgE, OVA-specific IgE, OVA-specific IgG1, and OVA-specific IgG2C levels in the serum of PBS- and bivalirudin TFA-treated mice were measured by ELISA (n=9,7 from left to right); the results are presented as the antibody titre extrapolated from a standard curve (if available) or as the absorbance at 450 nm (OD450 value) for the serum dilution for which all samples were in the range of the assay. (**c**) ELISA was performed to measure IL-5, IL-13 and IL-4 levels in the BALF (n=9,7 from left to right). (**d**) ELISA analysis of IL-5, IL-13, and IL-4 in the supernatants of lung lymphocytes from WT and α-MSH-treated mice (1×10^6 cells per well). Lymphocytes were stimulated with OVA extract for 4 days ex vivo (n=9,7 from left to right). (**e**) The numbers of ILC2s in the lungs (n=9,7 from left to right). (**f**) The numbers of IL-5+, IL-13+, and IL-5+ IL-13+ cells among lung ILC2s after stimulation with PMA, ionomycin, and BFA for 4 h (n=9,7 from left to right). Each symbol represents an individual mouse(**b,c,d,e,f**)The data are representative of two or more independent experiments. The bars and error bars show the means ± SDs. **b,c,d,e,f,** two-tailed unpaired Student's t test.

a.

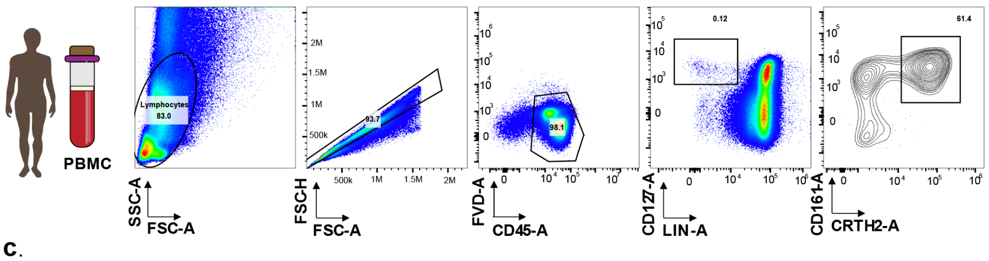

b.

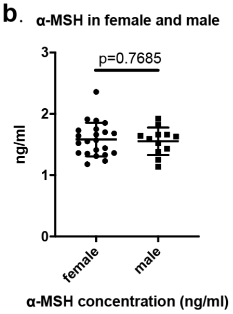

c.

ID	sex	BMI	ICS dose *IC	Comorbidity	ACQ score	FeNO（ppb）	IgE IU/ml	ILC2/ml blood	ILC2/CD45	IL5(pg/ml)	IL13(pg/ml)	Eos /ml blood	CD4/ml blood	a-MSH(ng/ml)
1	female	26.8	320	allergic rhinitis	1.428571429	86	51.1	318	0.110%	3119.6	1896.6	30413	167616	1.31
2	female	19.7	320	allergic rhinitis	1	58	89.2	378	0.048%	2724.0	1166.6	35048	202032	1.35
5	female	21.3	500	/	0.428571429	23	300.9	268	0.033%	173.5	500.2	148838	378047	1.68
7	female	25.7	160	allergic rhinitis	1.571428571	16	400.4	209	0.029%	793.2	1394.7	83700	248589	1.41
9	female	23.4	320	/	0.857142857	12	107.2	202	0.024%	1507.6	1895.0	124261	263675	1.55
10	female	22.4	320	allergic rhinitis	0.428571429	18	60.9	300	0.033%	835.7	1557.7	117990	253575	1.73
12	female	31.3	160	allergic rhinitis	1.142857143	5	187.8	832	0.088%	1555.0	1521.8	150552	361080	1.36
13	female	20.3	160	allergic rhinitis	0.714285714	19	162.2	46	0.005%	175.2	886.3	65274	355482	1.66
14	female	23.5	320	/	0.714285714	25	143.9	88	0.017%	176.8	842.3	44748	152098	1.64
21	female	17.4	320	allergic rhinitis	0.857142857	20	79.8	111	0.025%	444.9	1293.8	32412	126984	1.52
23	female	16.8	160	/	0.142857143	5	76.3	260	0.048%	100.2	1287.7	32273	265335	1.44
24	female	29.6	160	/	1	12	60.8	694	0.074%	506.9	1731.2	92581	388332	1.18
25	female	23.8	500	/	0.571428571	13	60.3	626	0.064%	592.6	1451.7	108558	349146	1.23
26	female	29.8	320	allergic rhinitis	0.571428571	16	49.9	333	0.032%	464.1	1706.9	99511	368868	1.75
28	female	25.6	500	allergic rhinitis	2	10	91.6	257	0.030%	577.6	1190.5	66924	235092	1.57
30	female	22.0	320	/	1	22	59.1	328	0.048%	519.8	710.5	46649	236664	2.36
32	female	23.3	320	allergic rhinitis	0.714285714	9	88.5	376	0.044%	714.6	1305.9	71053	252784	1.85
35	female	29.3	160	allergic rhinitis	0.714285714	73	49.7	276	0.023%	352.8	1093.3	114911	283672	1.89
36	female	17.4	320	allergic rhinitis	0.285714286	15	128.5	350	0.037%	275.8	1755.5	56192	370832	1.70
37	female	34.3	320	/	0.571428571	31	61	667	0.074%	506.9	1318.1	114554	394174	1.36
38	female	24.0	320	/	0.571428571	13	44.8	158	0.015%	151.6	1245.2	103714	364684	1.91
40	female	21.9	320	/	1.285714286	59	50.7	464	0.076%	671.8	1798.0	36173	244000	1.33
3	male	21.9	320	allergic rhinitis	0.285714286	75	46.7	117	0.023%	1519.0	1547.9	44848	125971	1.66
4	male	23.0	500	allergic rhinitis	0.428571429	20	180.1	167	0.029%	873.3	1112.8	76797	228388	1.64
8	male	25.1	160	/	1	30	103.8	214	0.017%	435.1	1055.8	86018	329522	1.63
11	male	34.0	320	coronary heart disease	0.428571429	20	70.3	115	0.021%	870.0	1138.9	37080	265680	1.38
17	male	29.1	320	/	1.142857143	30	401.5	260	0.041%	2164.8	1937.3	53852	328961	1.14
18	male	28.7	160	allergic rhinitis	1.714285714	20	68.5	35	0.010%	1038.4	1101.4	19629	120139	1.25
20	male	27.1	160	hypertension	1	22	69.5	186	0.071%	506.9	1251.2	33824	85215	1.43
22	male	26.9	160	allergic rhinitis	0.285714286	20	99.1	36	0.016%	440.6	1075.1	8026	39216	1.66
27	male	24.2	320	/	0.714285714	20	128.4	270	0.026%	521.9	1761.6	98384	322400	1.52
34	male	22.5	320	allergic rhinitis	2.142857143	12	62.6	240	0.045%	292.9	1427.4	12602	186900	1.59
39	male	24.2	320	allergic rhinitis	1	19	86.3	55	0.007%	1076.3	1299.8	51350	319160	1.92
41	male	23.7	320	/	0.714285714	21	77	146	0.024%	684.6	1190.5	45079	164700	1.82

Supplementary Figure 13. Asthma patient information.

(**a**) Gating strategy for human ILC2s among PBMCs. Human ILC2s were defined as $Lin^-CD45^+CD127^+CD161^+CRTH2^+$ cells. (**b**) α-MSH concentration in plasma of female and male asthma patients (female n=22, male n=12). Each symbol represents an individual patient. (**c**) The table displays data for asthma patient comorbidity, ACQ score, FeNO, IgE levels, eosinophil counts, $CD4^+$ T-cell counts, concentrations of α-MSH in plasma, numbers and percentages of ILC2s among live cells, and concentrations of IL-5 and IL-13 secreted by cultured PBMCs stimulated with IL-33 (patient age:18-70). The bars and error bars show the means ± SDs. **b**, two-tailed unpaired Student's t test.
