# Supplementary material for: Solar ultraviolet B radiation promotes α-MSH secretion to attenuate the function of ILC2s via the pituitary–lung axis
Source: Nat Commun. 2023 Sep 12;14:5601. doi: 10.1038/s41467-023-41319-1 (PMC10497598; doi:10.1038/s41467-023-41319-1)
Supplement: Supplementary file 3 — Reporting Summary [file 41467_2023_41319_MOESM3_ESM.pdf]

nature portfolio

Corresponding author(s): Bing Sun

Last updated by author(s): Jul 24, 2023

Reporting Summary

Nature Portfolio wishes to improve the reproducibility of the work that we publish. This form provides structure for consistency and transparency in reporting. For further information on Nature Portfolio policies, see our Editorial Policies and the Editorial Policy Checklist.

Statistics

For all statistical analyses, confirm that the following items are present in the figure legend, table legend, main text, or Methods section.

n/a	Confirmed	
☐	☒	The exact sample size (*n*) for each experimental group/condition, given as a discrete number and unit of measurement
☐	☒	A statement on whether measurements were taken from distinct samples or whether the same sample was measured repeatedly
☐	☒	The statistical test(s) used AND whether they are one- or two-sided<br>*Only common tests should be described solely by name; describe more complex techniques in the Methods section.*
☒	☐	A description of all covariates tested
☐	☒	A description of any assumptions or corrections, such as tests of normality and adjustment for multiple comparisons
☐	☒	A full description of the statistical parameters including central tendency (e.g. means) or other basic estimates (e.g. regression coefficient) AND variation (e.g. standard deviation) or associated estimates of uncertainty (e.g. confidence intervals)
☐	☒	For null hypothesis testing, the test statistic (e.g. *F*, *t*, *r*) with confidence intervals, effect sizes, degrees of freedom and *P* value noted<br>*Give P values as exact values whenever suitable.*
☒	☐	For Bayesian analysis, information on the choice of priors and Markov chain Monte Carlo settings
☒	☐	For hierarchical and complex designs, identification of the appropriate level for tests and full reporting of outcomes
☒	☐	Estimates of effect sizes (e.g. Cohen's *d*, Pearson's *r*), indicating how they were calculated

Our web collection on statistics for biologists contains articles on many of the points above.

Software and code

Policy information about availability of computer code

Data collection	Flow cytometry data were acquired with BD FACSDiva Software v8.0.2 or CytExpert2.4. qRT-PCR data were acquired with QuantStudio Real-Time PCR software v1.1. Sequencing was performed on an illumina NovaSeq 6000 platform. Histologic images were acquired using Zen2.3 software (Zeiss)
Data analysis	GraphPad Prism 8 was used to generate graphs and perform statistical analyses.<br>FlowJo V10 software for FACS analyses.<br>Image J 1.8 was used for western blot protein intensity analyses.

For manuscripts utilizing custom algorithms or software that are central to the research but not yet described in published literature, software must be made available to editors and reviewers. We strongly encourage code deposition in a community repository (e.g. GitHub). See the Nature Portfolio guidelines for submitting code & software for further information.

Data

Policy information about availability of data

All manuscripts must include a data availability statement. This statement should provide the following information, where applicable:

- Accession codes, unique identifiers, or web links for publicly available datasets
- A description of any restrictions on data availability
- For clinical datasets or third party data, please ensure that the statement adheres to our policy

Sequence data that support the findings of this study have been deposited in Genbank under the primary accession code[GSE237815] [GSE237962]. All other data are available in the article and supplementary files or from the corresponding authors upon reasonable request. The source data and uncropped immunoblot images are included in the Source Data files

Human research participants

Policy information about studies involving human research participants and Sex and Gender in Research.

Reporting on sex and gender	The sex and gender are described in supplementary Figure 7b and human samples of material methods.
Population characteristics	Human blood samples were obtained from male and female asthmatic participants (age 18 to 70) .
Recruitment	The average emergency asthma (ED) visit counts for each day between 2015 and 2019 were obtained from Ruijin Hospital, Huashan Hospital, Dongfang Hospital, Shanghai Fifth People's Hospital, Shanghai Putuo District Central Hospital, and Shanghai Putuo District People's Hospital.<br>Asthmatic patients were recruited from Ruijin Hospital, Shanghai Jiaotong University School of Medicine. The diagnosis of asthma was established based on respiratory symptoms and evidence of variable airflow obstruction according to the Global Initiative for Asthma (GINA) guidelines. A lung function test was performed, and the predicted forced expiratory volume in 1 s (FEV1) percent (FEV1% pre), FEV1/forced vital capacity percentage (FEV1/FVC), disease duration and asthma control questionnaire (ACQ) scores were recorded. Patients with an acute attack, autoimmune diseases, or infectious diseases and those who had received systemic glucocorticoids and immunosuppressive agents within 1 month before the study were excluded.<br>All patients who provided informed consent had sample collected;all study procedures were conducted in strict compliance with ethical and institution regulations.
Ethics oversight	This study was approved by the Research Ethics Board of Ruijin Hospital, Shanghai Jiao Tong University School of Medicine (2022-187)(2021-63).

Note that full information on the approval of the study protocol must also be provided in the manuscript.

Field-specific reporting

Please select the one below that is the best fit for your research. If you are not sure, read the appropriate sections before making your selection.

☒ Life sciences ☐ Behavioural & social sciences ☐ Ecological, evolutionary & environmental sciences

For a reference copy of the document with all sections, see nature.com/documents/nr-reporting-summary-flat.pdf

Life sciences study design

All studies must disclose on these points even when the disclosure is negative.

Sample size	Sample size was chosen based on prior knowledge from previous experiments. Key experiments were repeated by 2 independent researchers. In the in vivo assays,the lung tissues from 3-8 mouse per group in this study were analyzed to ensure the differences. In the in vitro assays,variability used in this study tends to be low,so n>=3 is accepted.
Data exclusions	No data was excluded.
Replication	All experiments were performed at least two or three times as independent experiments and were successfully replicated. This is indicated in the figure legends for each experiments.
Randomization	Age and sex-matched mice were randomly allocated to experimental group. Randomization process consisted of pooling all mice in a large cage and picking out and allocating to experimental groups randomly. All human subjects were randomly allocated to different groups.
Blinding	No blinding were used as the design and conditions prevent possible sources of bias.

Reporting for specific materials, systems and methods

We require information from authors about some types of materials, experimental systems and methods used in many studies. Here, indicate whether each material, system or method listed is relevant to your study. If you are not sure if a list item applies to your research, read the appropriate section before selecting a response.

Materials & experimental systems

n/a	Involved in the study	
☐	☒	Antibodies
☒	☐	Eukaryotic cell lines
☒	☐	Palaeontology and archaeology
☐	☒	Animals and other organisms
☒	☐	Clinical data
☒	☐	Dual use research of concern

Methods

n/a	Involved in the study	
☒	☐	ChIP-seq
☐	☒	Flow cytometry
☒	☐	MRI-based neuroimaging

Antibodies

Antibodies used

flow cytometry antibody:
anti-mouse CD45R-APC (BioLegend,RA3-6B2, 103212, 1/100)
anti-mouse CD5-APC (BioLegend,53-7.3, 100626, 1/100)
anti-mouse NK1.1-APC (BioLegend, PK136, 108710, 1/100)
anti-mouse TCR γ/δ-APC (BioLegend, UC7-13D5, 118116, 1/100)
anti-mouse Gr-1-APC (BioLegend, RB6-8C5, 108412, 1/100),
anti-mouse erythroid cell marker TER119-APC (BioLegend, TER-119, 116212, 1/100),
anti-mouse CD45.2-Pacific Blue (BioLegend, 104, 109820, 1/150),
anti-mouse Thy1.2- PerCP/Cyanine5.5 (BioLegend, 53-2.1, 140322, 1/150)
anti-mouse CD25-Brilliant Violet 421 (BioLegend, PC61, 102034, 1/100)
anti-mouse IL-5-Brilliant Violet 421 (BioLegend, TRFK5, 504311, 1/100)
anti-mouse p-STAT3-Brilliant Violet 421 (BioLegend, 13A3-1, 651010, 1/100)
anti-mouse CD3-APC (eBioscience, 2C11, 17-0031-83, 1/100)
anti-mouse CD11b-APC (eBioscience, M1/70, 17-0112-83, 1/200)
anti-mouse CD11c-APC (eBioscience, N418, 17-0114-82, 1/400)
anti-mouse FcεR1-APC (eBioscience, MAR-1, 17-5898-82, 1/200)
anti-mouse IL-13-PE (eBioscience, eBio13A, 12-7133-82, 1/100)
anti-mouse KLRG1- Pacific Blue (eBioscience, 2F1, 48-5893-82, 1/100)
anti-mouse SCA-1-PE-Cyanine7 (eBioscience, D7, 25-5981-81, 1/100)
anti-mouse C-KIT-PerCP-eFluor™ 710 (eBioscience, 2BB, 46-1171-82, 1/100)
anti-mouse CD16/CD32 (eBioscience, 93,14-0161-86, 1/100)
anti-mouse ki67-A700(eBioscience, SolA15,56-5698-82, 1/100)
anti-mouse CD19-FITC(eBioscience, eBio1D3,11-0193-85, 1/100)
anti-mouse CD103-APC(eBioscience, 2E7,17-1031-82, 1/100)
anti-mouse MHC-II-APC-eFluor 780(eBioscience, M5/114.15.2,47-5321-82,1/100)
anti-mouse CD127-PE-cy7 (BD Pharmingen, A7R34, 560733, 1/100)
anti-mouse Siglec-F-APC (BD Pharmingen,E50-2440, 562680, 1/100)
anti-mouse p-STAT5-Alexa Fluor 647 (BD Pharmingen,47/Stat5, 562076, 1/100)
anti-mouse IL-33R-FITC (MD Bioproducts,DJ8, 101001F, 1/100)
anti-human CD127-PE (BioLegend, A019D5, 351304, 1/100)
anti-human CD161-APC/Cyanine7 (BioLegend, HP-3G10, 339928, 1/100)
anti-human CRTH2-FITC (BioLegend, BM16, 350108, 1/100)
anti-human CD45-PerCP/Cyanine5.5 (BioLegend, HI30, 304028, 1/100)

Elisa antibody:
anti-mouse IgE (BD pharmingen, capture antibody; 553413; 1/250)
Goat Anti-Mouse IgE-HRP (SouthernBiotech detection antibody; 1110-05; 1/16000)
HRP Rat Anti-Mouse IgG1 (BD pharmingen; 559626; 1/250)
Goat Anti-Mouse IgG2c-HRP (SouthernBiotech; 1078-05; 1/4000)

WB antibody:
Rabbit anti-mouse CRH(Abcam,ab184238,1/5000)
Rabbit anti-mouse POMC(Abcam, ab210605, 1/5000)
Rabbit anti-mouse p-STAT3(CST,9131s,1/1000)
Rabbit anti-mouse p-STAT5(CST,9359s,1/1000)
Rabbit anti-mouse p-p65(CST,3033s,1/2000)

Beta Actin Monoclonal antibody(Proteintech, 66009-1-Ig,1/5000)
Goat Anti-Rabbit IgG HRP Affinity Purified PAb (R&D Systems, HAF008,1/1000)
Goat Anti-mouse IgG HRP Affinity Purified PAb (R&D Systems, HAF007,1/1000)

Immunofluorescence antibody:
rabbit anti-CRH (Abcam, ab272391, 1/200)
rabbit anti-POMC (Abcam, ab210605, 1/500)
goat anti-rabbit CY3 (Servicebio, GB21303, 1/300)

Validation

fixable viability stain 780 (BD Pharmingen, 565388,1/1000)
(App:FC React:Mammalian cells)

anti-mouse CD45R-APC (BioLegend,RA3-6B2, 103212, 1/100)
(App:FC React:mouse,human)

anti-mouse CD5-APC (BioLegend,53-7.3, 100626, 1/100)
(App:FC React:mouse)

anti-mouse NK1.1-APC (BioLegend, PK136, 108710, 1/100)
(App:FC React:mouse)

anti-mouse TCR γ/δ-APC (BioLegend, UC7-13D5, 118116, 1/100)
(App:FC React:mouse)

anti-mouse Gr-1-APC (BioLegend, RB6-8C5, 108412, 1/100)
(App:FC React:mouse)

anti-mouse erythroid cell marker TER119-APC (BioLegend, TER-119, 116212, 1/100)
(App:FC React:mouse)

anti-mouse CD45.2-Pacific Blue (BioLegend, 104, 109820, 1/150)
(App:FC React:mouse)

anti-mouse Thy1.2- PerCP/Cyanine5.5 (BioLegend, 53-2.1, 140322, 1/150)
(App:FC React:mouse)

anti-mouse CD25-Brilliant Violet 421 (BioLegend, PC61, 102034, 1/100)
(App:FC React:mouse)

anti-mouse IL-5-Brilliant Violet 421 (BioLegend, TRFK5, 504311, 1/100)
(App:FC React:mouse,human)

anti-mouse p-STAT3-Brilliant Violet 421 (BioLegend, 13A3-1, 651010, 1/100)
(App:ICFC React:mouse,human)

anti-mouse CD3-APC (eBioscience, 2C11, 17-0031-83, 1/100)
(App:FC React:mouse)

anti-mouse CD11b-APC (eBioscience, M1/70, 17-0112-83, 1/200)
(App:FC React:mouse)

anti-mouse CD11c-APC (eBioscience, N418, 17-0114-82, 1/400)
(App:FC React:mouse)

anti-mouse FcεR1-APC (eBioscience, MAR-1, 17-5898-82, 1/200)
(App:FC React:mouse)

anti-mouse IL-13-PE (eBioscience, eBio13A, 12-7133-82, 1/100)
(App:FC React:mouse)

anti-mouse KLRG1- Pacific Blue (eBioscience, 2F1, 48-5893-82, 1/100)
(App:FC React:mouse)

anti-mouse SCA-1-PE-Cyanine7 (eBioscience, D7, 25-5981-81, 1/100)
(App:FC React:mouse)

anti-mouse C-KIT-PerCP-eFluor™ 710 (eBioscience, 2BB, 46-1171-82, 1/100)
(App:FC React:mouse,pig)

anti-mouse CD16/CD32 (eBioscience, 93,14-0161-86, 1/100)
(App:FC,Neu,FN React:mouse)

anti-mouse ki67-A700(eBioscience, SolA15,56-5698-82, 1/100)
(App:FC React:mouse,dog,Cynomolgus monkey, Human,Non-human primate, Rat)

anti-mouse CD19-FITC(eBioscience, eBio1D3,11-0193-85, 1/100)
(App:FC React:mouse)

anti-mouse CD103-APC(eBioscience, 2E7,17-1031-82, 1/100)
(App:FC React:mouse)

anti-mouse MHC-II-APC-eFluor 780(eBioscience, M5/114.15.2,47-5321-82,1/100)
(App:FC React:mouse)

anti-mouse CD127-PE-cy7 (BD Pharmingen, A7R34, 560733, 1/100)
(App:FC React:mouse)

anti-mouse Siglec-F-APC (BD Pharmingen,E50-2440, 562680, 1/100)
(App:FC React:mouse)

anti-mouse p-STAT5-Alexa Fluor 647 (BD Pharmingen,47/Stat5, 562076, 1/100)
(App:FC React:mouse,human)

anti-mouse IL-33R-FITC (MD Bioproducts,DJ8, 101001F, 1/100)
(App:FC React:mouse)

anti-human CD127-PE (BioLegend, A019D5, 351304, 1/100)
(App:FC React:human)

anti-human CD161-APC/Cyanine7 (BioLegend, HP-3G10, 339928, 1/100)
(App:FC React:human)

anti-human CRTH2-FITC (BioLegend, BM16, 350108, 1/100)
(App:FC React:human)

anti-human CD45-PerCP/Cyanine5.5 (BioLegend, HI30, 304028, 1/100)
(App:FC React:human)

anti-mouse IgE (BD pharmingen, capture antibody; 553413; 1/250)
(App:ELISA Capture React:mouse)

Goat Anti-Mouse IgE-HRP (SouthernBiotech detection antibody; 1110-05; 1/16000)
(App:ELISA detect,FC,IHC,WB,IP React:heavy chain of mouse)

HRP Rat Anti-Mouse IgG1 (BD pharmingen; 559626; 1/250)
(App:ELISA React:mouse)

Goat Anti-Mouse IgG2c-HRP(SouthernBiotech; 1078-05; 1/4000)
(App:ELISA React: heavy chain of C57BL/6 mouse IgG2c)

Rabbit anti-mouse CRH(Abcam,ab184238,1/5000)
(App:WB React: mouse, rat, human)

Rabbit anti-mouse POMC(Abcam, ab210605, 1/5000)
(App:WB,IP,IHC-P React: mouse, rat, human,Recombinant fragment)

Rabbit anti-mouse p-STAT3(CST,9131s,1/1000)
(App:WB,IP React:mouse,human,rat,monkey)

Rabbit anti-mouse p-STAT5(CST,9359s,1/1000)
(App:WB,IP,IHC,FC React:mouse,human)

Rabbit anti-mouse p-p65(CST,3033s,1/2000)
(App:WB,IP,IF,FC React:mouse,human,rat,hamster,milk,monkey,pig)

Beta Actin Monoclonal antibody(Proteintech, 66009-1-Ig,1/5000)
(App: FC, IF, IHC, IP, WB, ELISA React:Human, Mouse, Rat, Hamster, Monkey, Dog, Pig, Chicken, Rabbit, Zebrafish)

Goat Anti-Rabbit IgG HRP Affinity Purified PAb (R&D Systems, HAF008,1/1000)
(App:WB, IHC React:rabbit)

Goat Anti-mouse IgG HRP Affinity Purified PAb (R&D Systems, HAF007,1/1000)
(App:WB, IHC React: mouse)

rabbit anti-CRH (Abcam, ab272391, 1/200)
(App:IHC,IF React: mouse human)

rabbit anti-POMC (Abcam, ab210605, 1/500)
(App:WB,IP,IHC-P React: mouse, rat, human,Recombinant fragment)

goat anti-rabbit CY3 (Servicebio, GB21303, 1/300)
(App:IF React: rabbit)

Animals and other research organisms

Policy information about studies involving animals; ARRIVE guidelines recommended for reporting animal research, and Sex and Gender in Research

Laboratory animals	C57BL/6 mice were purchased from the Shanghai Research Center for Model Organization (Shanghai, China). Rag1−/− mice were maintained at the Chinese Academy of Sciences (Shanghai, China). Mc5rfl/fl mice were purchased from GemPharmatech (Nanjing, China). Il5tm1.1 (icre)Lky/J(R5/+) mice were ordered from Jackson laboratory. Mice were maintained at macroenvironmental temperature of 21–22 °C, humidity (48–52%), in a conventional 12:12 light/dark cycle with lights on at 6:00 a.m. and off at 6:00 p.m. Mice were maintained under specific pathogen-free conditions at the Animal Care Facility of the Chinese Academy of Sciences (Shanghai, China) and used at the age of 6-8 wk. The animal care and use procedures complied with the guidelines of the Institute of Biochemistry and Cell Biology, Chinese Academy of Sciences.
Wild animals	The study did not involve wild animals.
Reporting on sex	The findings don't apply to only one sex.
Field-collected samples	The study did not involve samples collected from the field.
Ethics oversight	All animal experiments were approved by the Institutional Animal Care and Use Committee of the Shanghai Institute of Biochemistry and Cell Biology, Chinese Academy of Sciences (ethics approval no. SIBCB-S303-1610-030-c2).

Note that full information on the approval of the study protocol must also be provided in the manuscript.

Flow Cytometry

Plots

Confirm that:

- [x] The axis labels state the marker and fluorochrome used (e.g. CD4-FITC).
- [x] The axis scales are clearly visible. Include numbers along axes only for bottom left plot of group (a 'group' is an analysis of identical markers).
- [x] All plots are contour plots with outliers or pseudocolor plots.
- [x] A numerical value for number of cells or percentage (with statistics) is provided.

Methodology

Sample preparation	Mice were euthanized by CO_2, and BALF cells were collected from the lungs by gentle washing with 800 µl of ice-cold PBS twice with 18-G plastic cannulas and 1-ml syringes. For the isolation of lung cells, mice were perfused with 10 ml of cold DPBS via the right heart ventricle to remove blood from the lungs. Removed lungs were minced with scissors. Then, the minced lung tissue was placed in RPMI-1640 medium containing 10% foetal calf serum (FCS, Thermo Fisher) and 1% penicillin–streptomycin (Cytiva HyClone, SV30010) and digested with 0.5 mg/ml collagenase type I (Invitrogen, 17100017) for 30 min at 37°C with continuous agitation in an incubator. The digested lung fragments were passed through a 70-µm filter to isolate the cell suspension and centrifuged at 350 × g for 10 min. The red blood cells were lysed in BD Pharm lyseTM lysis buffer (BD Pharmingen, 555899). The supernatant was aspirated completely. The pellets were suspended in 40% Percoll (GE Healthcare, 17089109), which was layered over 80% Percoll; the gradient was centrifuged at 600 × g for 20 min to obtain lung mononuclear cells, and the isolated cells were washed with RPMI-1640 medium before further analysis.
Instrument	LSRFortessa (BD), CytoFlex3 (Beckman).
Software	FlowJo software version10.6
Cell population abundance	Cell sorting gating strategy of lung ILC2:Mouse lung monocytes were firstly identified by gating on lymphocytes and then

	remove doublets and aggregated particles for single cells. Live cells were circled and then identify Lin-CD127+CD90.2+ST2+ cells in CD45+ population. The purity of the obtained ILC2 were 99%-100%
Gating strategy	Mouse lung ILC2s (CD45+Lin−CD127+CD90.2+ST2+);mouse eosinophils(FVD-CD45+CD11c-SiglecF+);human ILC2s (FVD-CD45 +LIN-CD161+CD127+CRTH2+);B cells(FVD-CD3-CD19+);CD4+cells (FVD-CD3+CD4+);CD45+cells(FVD-CD45+); CD8+cells(FVD-CD3-CD8+);DCs(FVD-CD45+CD103+MHC-II+CD11C+); Alveolar macrophage(AMs, FVD-CD45+Ly6G-CD11C+SiglecF+) Interstitial macrophages(IM: CD45+Ly6G−SiglecF−CD11c+CD11b+F4/80+);Neurophils(FVD-CD45+Ly6G+CD11B+); NK cells (FVD-CD45+CD3−NKp46+).

☒ Tick this box to confirm that a figure exemplifying the gating strategy is provided in the Supplementary Information.
